# Effect of different cross sections of polyetheretherketone splinting bars in maxillary All-on-4^®^ implant supported interim prosthesis: finite element analysis study

**DOI:** 10.1186/s12903-025-06542-w

**Published:** 2025-07-28

**Authors:** Rana Mohammad Abdelrahman, Ahmed Mostafa Abdelfattah, Marwa Kothayer

**Affiliations:** 1 Oral and Maxillofacial Prosthodontics Department, School of Dentistry, Newgiza University, Giza, Egypt; 2 Oral and Maxillofacial Prosthodontics Department, Faculty of Dentistry, AinShams University, Organization of African Unity Street, Cairo, 11561 Egypt

**Keywords:** Implant splinting, Stress analysis, Interim prosthesis fracture

## Abstract

**Background:**

All-on-4^®^ concept allows the immediate rehabilitation of edentulous arches having anatomical limitations with provisional restoration. However, the acrylic provisional prosthesis has a critical liability to fracture, that’s why a reinforcement with splinting bar is a good solution for a longer-term success. Poly Ether Ether ketone has been used as a splinting material since they are biocompatible with an elastic modulus similar to native bone and dentin with shock-absorbing characteristics. The purpose of this study was to evaluate the effect of different cross sections of Poly Ether Ether ketone splinting bars in maxillary All-on-4^®^ implant supported interim prosthesis.

**Methods:**

The current study involved two steps: virtual model construction and three-dimensional Finite Element Analysis. The virtual model composed of an edentulous maxillary cast, four dental implants placed according to the All-on-4^®^concept and an acrylic prosthetic superstructure reinforced with Poly Ether Ether ketone bar. Two virtual models were made. In the virtual model RB, a Poly Ether Ether ketone bar with rounded cross section was used. However, in the virtual model SB, a square bar was used. Each model was subjected to two types of loads: vertical load and oblique load. Each model was analyzed, and the stresses displayed in the current study are the von-Mises stress (SEqv) for the implants, marginal part of the bone cylinder, Poly Ether Ether ketone bar, prosthesis and the entire model.

**Results:**

Higher stress values were observed in the model RB compared to the model SB in the implants, peri-implant bone and overlying prosthetic superstructure. However, higher stress values were recorded for the splinting bar in the model SB compared to the RB one. The highest recorded value in model RB was 120.26 MPa^*^ which recorded in the prosthetic superstructure during unilateral oblique loading while the highest recorded value in model SB model was 110.51 MPa^*^ which recorded in the posterior implant during unilateral oblique loading.

**Conclusion:**

Square cross sectioned Poly Ether Ether ketone bar induce less stress in All-On-4^®^ Implant Supported Interim Prosthesis compared to the round one.

## Background

Prosthetic rehabilitation of totally edentulous patients using dental implants is considered one of the most common approaches nowadays [1] It results in many improvements regarding the rate of bone resorption, retention, and support of the prostheses, as well as the improved functions. Eventually this leads to an overall better quality of life and patient satisfaction [2].

The idea of all-on-4 implants was first introduced by Maló in the late nineties. This concept allows the immediate rehabilitation of edentulous arches having anatomical limitations with provisional restoration [3]. It highly meets patient satisfaction and answers the concerns that revolve around the esthetics, phonetics, and comfort [4].

Fracture of the provisional acrylic prosthesis was the most common reported problem in All-on-Four concept according to a a systematic review evaluated the survival rates of axial and tilted implants in the rehabilitation of edentulous jaws utilizing the All-on-Four concept. Wear patterns in the opposing dentition and prosthetic screw loosening were other problem noted in patients with bruxism. Additionally, they found that there was no significant difference in the outcome of tilted versus axial implants in the maxilla and the mandible [5].

Since the acrylic provisional prosthesis has a critical liability to fracture, that’s why a reinforcement with splinting bar is a good solution for a longer-term success than just during the healing process [6].

One main requirement needed during the interim phase is to establish a good primary stability of the dental implants [7–10]. The idea of implant splinting has been proven success in maintaining the mechanical stability of the implants after surgery [11–13]. Therefore, many authors have adopted the use of titanium bars for splinting the implants in the edentulous jaws, whereas the prefabricated bar is first modelled to fit the abutments, and then welded directly inside the oral cavity. Welding the implants together leads to better stress distribution as the implants no longer act individually, but rather, share in providing the mechanical support for the prosthesis [13, 14].

On the other hand, it can be difficult to find the best passive contacts for the titanium bar with the abutments during the process of intra-oral welding; an important condition to limit the micro-movements and provide a rigid, passive framework splinting [15]. Another drawback from a biomechanical perspective, is that the titanium bar fixation could induce torsional stresses that may be transmitted to the prosthetic superstructure resulting in fracture and failure of the overlying interim prosthesis [13]. Also, titanium-based materials can form a heat affected zone during welding procedures, in addition to the high reactivity of the titanium with oxygen, nitrogen and hydrogen at high temperatures [16].

To overcome all these downsides, polymers, such as, Poly Ether Ether ketone (PEEK) have been used as a splinting material since they are biocompatible with an elastic modulus similar to native bone and dentin with shock-absorbing characteristics. Moreover, they are inert and non-allergenic polymeric biomaterials. Additionally, they have good mechanical properties, high temperature resistance, chemical stability, polishability, and most importantly, they can be easily obtained in three dimensional forms [17–19].

Regina et al. [20] have studied the mechanical response of PEEK and PEKK (polyetherketoneketone) as frameworks for full-arch prostheses following the all-on-four concept, and they have concluded that PEKK has a lower stress concentration on the prosthetic screw and prosthetic base, meanwhile, lower stress concentration was observed on PEEK frameworks. Amanda et al. [21] evaluated the flexural strength of bars on implants made of PEEK and metal, and they used three different cross-sectional designs: rectangular; T-shaped bar; and inverted-T shaped bar. They concluded that the bar design influences the flexural strength, and metal bars showed higher flexural strength relative to PEEK.

The design of the PEEK bar can minimize flaws. As a larger crosssectional area prevents fractures, this conclusion was reached as well by the same study conducted by Amanda et al. [21], where different designs of PEEK bars were compared. The rectangular bars displayed superior compression strength results, followed by Ttype and inverted Ttype. The poor performance of the inverted Ttype bars was possibly caused by the smaller contact area that created a fulcrum on which the stress was concentrated.

In a finite element analysis study done by Kupprano et al. [22], four different geometric cross-sectional bars were used for a mandibular implant supported hybrid prosthesis following the “All-on-4” concept. L, Y, I, and Square cross sectional bars of equal volume, were created. It was concluded in this study that different cross-sectional designs provide varying levels of flexural strength and resistance to deformation, and it was found that the Y model demonstrated the lowest stress level and the least bending.

Another study by Jennifer et al., investigated experimentally and numerically the fracture and fatigue behavior of three different materials (titanium, cobalt chromium, and PEKK) when used as implant supported bars with distal extension. They determined that PEKK could be considered a promising material and a good alternative for patients with metal allergy although, higher deformation that occurred would limit the intraoral long-term stability compared to metal bars [23].

Finite-element analysis, with all its limitations, still presents a valuable instrument allowing us to realize the nature of stress and strain distribution in bone tissue and implants, and how they are affected by the restorative material, framework design, the prosthesis and manufacturing technique [24, 25]. Also, it offers several advantages, as the accurate representation of complex geometries, easy model modification, and other mechanical quantities [26].

Ouldyerou et al. used a finite element analysis to evaluate the biomechanical effect of implant length, marginal bone loss levels, and load magnitude under axial and buccolingual loading on stress at the bone–implant interface. Marginal bone loss still a controversial problem and a major complication in the field of implantology. It was concluded that progressive marginal bone loss and excessive loading produce higher stresses in the bone, which may disrupt the bone remodeling process [27].

Almjaddr et al. studied the distribution of stress in All-on-4 mandibular prostheses in the bone, implants, and framework according to difference cantilever length in PEEK prosthetic framework using three-dimensional finite element analysis. The study found that a cantilever length of 10 mm resulted in increased stress compared to a cantilever length of 15 mm in the cortical bone, the implants and framework. However, both PEEK models demonstrated a similar stress distribution in the spongy bone across both cantilever lengths [28].

Mohamed et al. had presented a finite element analysis study that examined the stresses induced by one-piece and two-piece dental implants in All-on-4 concept when subjected to lateral load [29]. They concluded that the one-piece dental implant induced less stresses compared to the two-piece implant. Also, a finite element analysis study by Otávio et al. had evaluated the biomechanical behavior of PEEK bar in all-on-four implant prosthesis against nickel–chromium bar under axial and lateral load [30]. A conclusion was reached that the overall performance of PEEK bar is favorable, although larger stress transference was observed in PEEK bar under unilateral load. So, the purpose of the current study was to evaluate the difference in the stresses induced by different cross sections of Polyetheretherketone splinting bars; round and square ones in maxillary All-on-4^®^ implant supported interim prosthesis using Finite Element Analysis. The null hypothesis for the current study was that no difference existed between both bar cross sections.

## Methods

The current study involved two steps: virtual model construction and three-dimensional Finite Element Analysis. The virtual model composed of an edentulous maxillary cast, four dental implants placed according to the All-on-4^®^concept and an acrylic prosthetic superstructure reinforced with PEEK bar. Two virtual models were made. In the virtual model RB, a PEEK bar with round cross section was used. However, in the virtual model SB, a square bar was used.

**Fig. 1:**
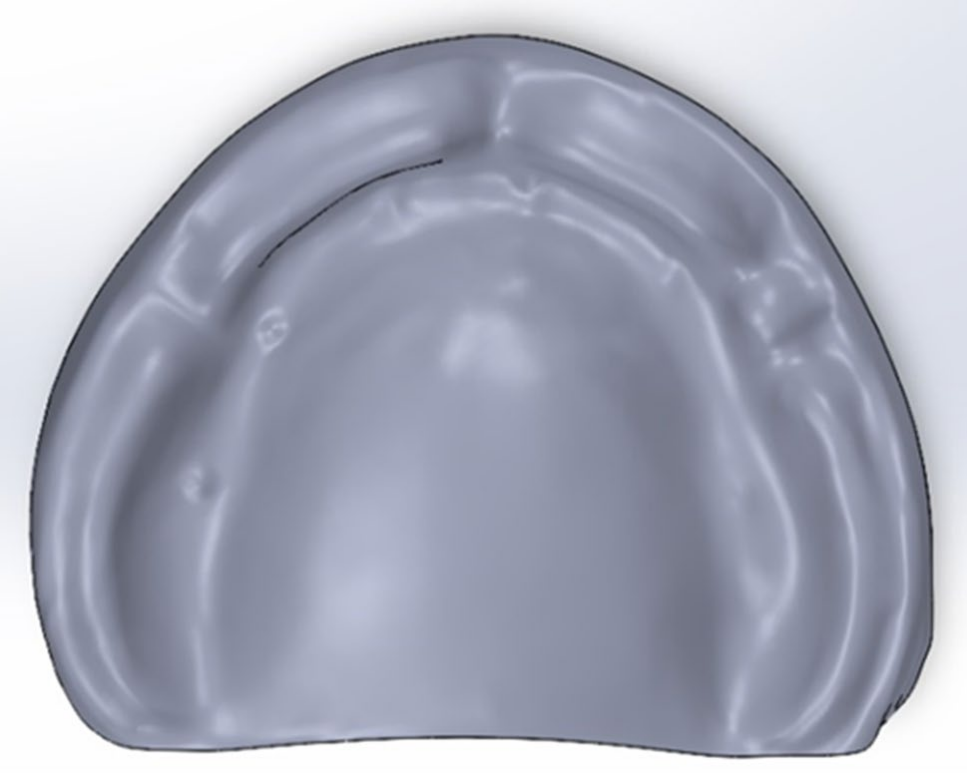
A 3D solid body of the maxillary cast

For the virtual model construction, an educational maxillary edentulous cast (Ramses medical products, Cairo, Egypt) was used. It was scanned using a desktop scanner (CeraMap 400 Amann Girrbach America Inc., Koblach, Austria) and modelled using a CAD software (Autodesk Meshmixer software, San Rafael, California, United States of America) to make a solid cast using the “make solid” feature of the software. Using the “Mesh2surface add-in” in the Solidworks software (Solidworks2020 SolidWorks Corp., Dassault Systèmes, Villacoublay, France), reverse engineering was done to convert the STL form of the cast into a simple 3D solid body form of the cast (Fig. 1) [29, 31].

**Fig. 2:**
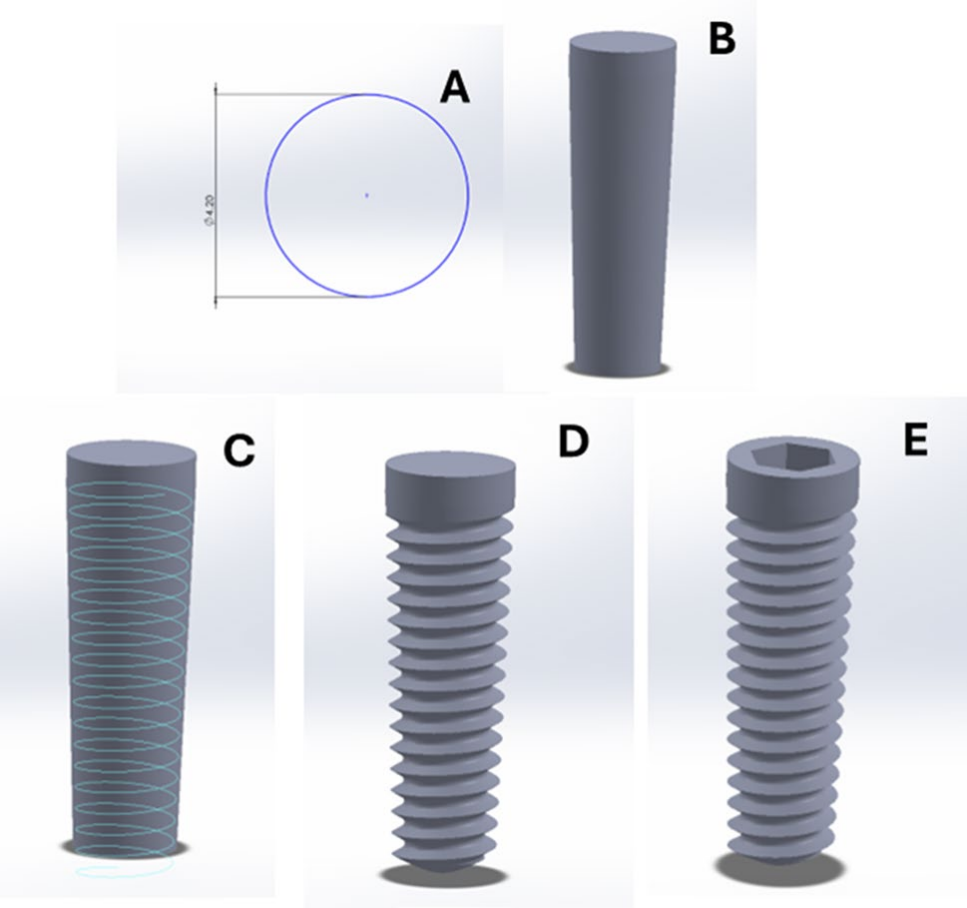
A 2D sketch of a circle in the top plane for implant modelling (**A**), a cylinder representing the implant body (**B**), an implant threads modelling with a helix (**C**), the cross section of the threads, and cutting them (**D**) the creation of the cavity for the threaded multiunit abutment (**E**)

Four dental implants (Flotechno dental implants, Milano, Italy) were planned to be placed in the anterior and posterior regions following the All-on-4^®^concept. The anterior implants (4.2*11 mm) were axially placed in the lateral incisor region while the posterior ones (4.2*13) were placed in the premolar region with a distal angulation 45 degrees. The implants were modelled using the Solidworks software (SolidWorks Corp., Dassault Systèmes, Villacoublay, France) as a 2D sketch of a 4.2 mm diameter circle that was drawn in the top plane (Fig. 2A). The circle was then extruded using the “boss extrude” tool to form a cylinder of 11 mm and 13 mm length for the anterior and posterior implants respectively with a taper of 2 degrees with a regular platform matching implant of 4 mm diameter (Fig. 2B). For implant threads modelling, a helix having 0.7 mm pitch distance, and 2 degrees taper angle was made along the length of the implant cylinder using the “Helix/spline” tool (Fig. 2C). The cross section of the threads was drawn, and the threads were then cut using the “Sweep cut” tool (Fig. 2D). A cavity for the threaded multiunit abutment was created using the “insert a cavity” tool, in which the multiunit abutment was inserted into the implant (Fig. 2E) [29, 31].

A bone cylinder was designed around each implant to facilitate data collection especially at the marginal part. The length of the cylinder varied according to the length of the implant; 13 mm and 15 mm for the anterior and posterior implants respectively. For designing the bone cylinder, a circle was drawn and extruded according to the previously mentioned lengths using the “Boss extrude” command. The cervical part of each bone cylinder was assumed to be compact bone. On the other hand, the remaining part of the bone cylinder was assumed to be cancellous bone. The implant was inserted into the bone cylinder and its place was indented into the bone using the cavity feature [29, 31].

**Fig. 3:**
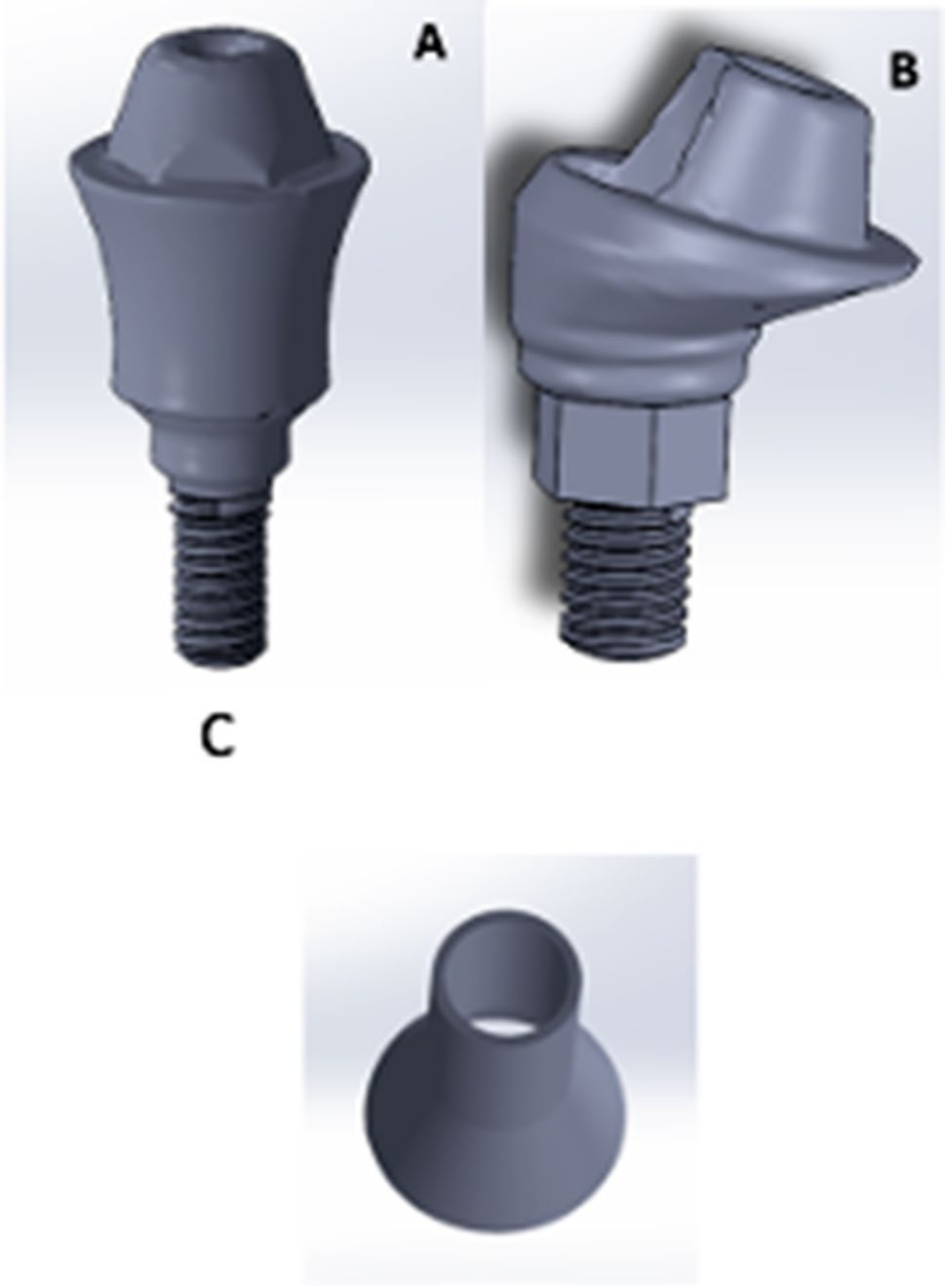
A 3D model of the straight multiunit abutment (**A**), a 3D model of the angled multiunit abutment (**B**), a 3D hollow Titanium cylinder cavity (**C**)

The Straight and angled multiunit abutments were exported as STL file from BlueSky Bio software. The STL file was then exported to the Solidworks software and reversed engineered to form a 3D object (Fig. 3A and B). While for the titanium cylinders modelling, a 2D sketch of a 5 mm diameter circle was drawn in the top plane over the multiunit abutment. The circle was extruded using the “boss extrude” tool to form the tapered cap part of 2.5 mm length that would fit on the multiunit abutment. Finally, a cavity was made inside the cap part and the cylindrical part to form the final 3D hollow Titanium cylinder 5 mm length (Fig. 3C) [29, 31].

**Fig. 4:**
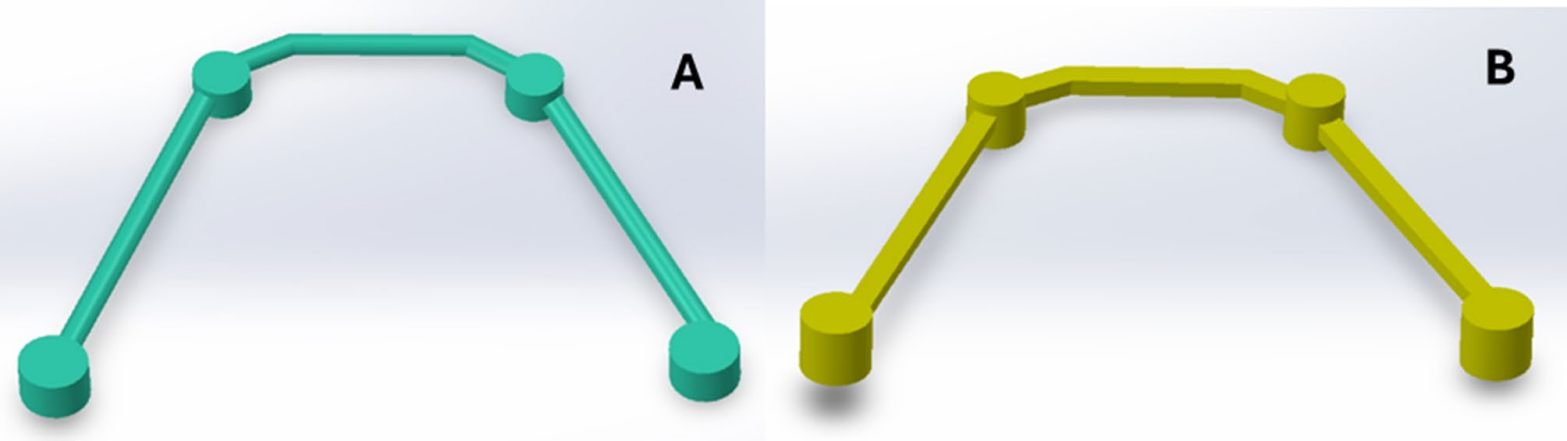
A photo showing the round (**A**) and the square (**B**) cross sections of the PEEK bar designed by The “swept base” command

For the PEEK bar modeling, A 2D sketch was drawn to determine the path of the bar over the implants. The bar framework was formed using the “swept base” command to have a cross section of 2.5 mm thickness. Holes were then made in the framework corresponding to the site of the titanium cylinders. The “swept base” command was also used to design the round and square cross sections of the PEEK bar in the models RB and SB respectively (Fig. 4).

The STL file of the scanned maxillary cast was exported to the Exocad software (Exocad America, Inc. Darmstadt, Germany) and the prosthetic superstructure was designed including the teeth and the underlying gingival part. The design was then exported as STL file to Solidworks software for reverse engineering using the “Mesh2surface” to give the simple 3D object [29, 31].

**Fig. 5:**
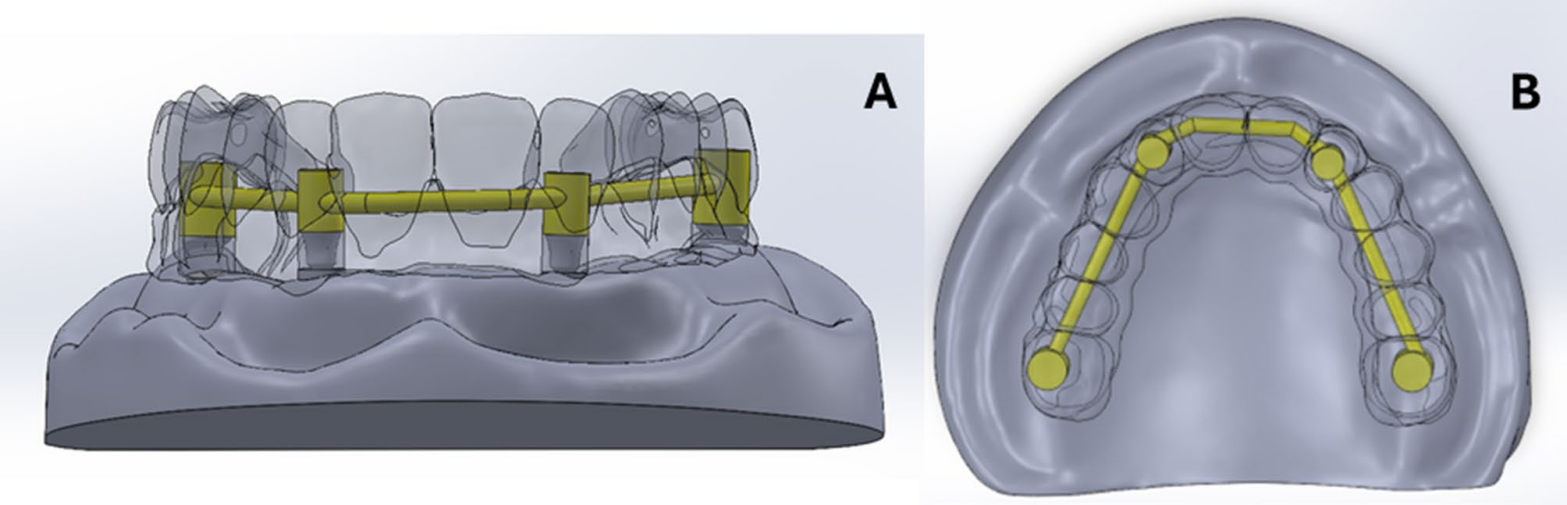
The model showing the assembly of the components (**A**: frontal view **B**: occlusal view)

The SolidWorks software was used for components assembly. The components were assembled with each other using the “Mating” tool of the software to place the components in the correct position based on the point of origin between the different parts. Care was taken to assemble the parts together without interference by making a cavity in each part with the overlying component using the “Cavity Feature” tool of the software. Interference was also checked by the interference detection tool (Fig. 5) [29, 31].

**Fig. 6:**
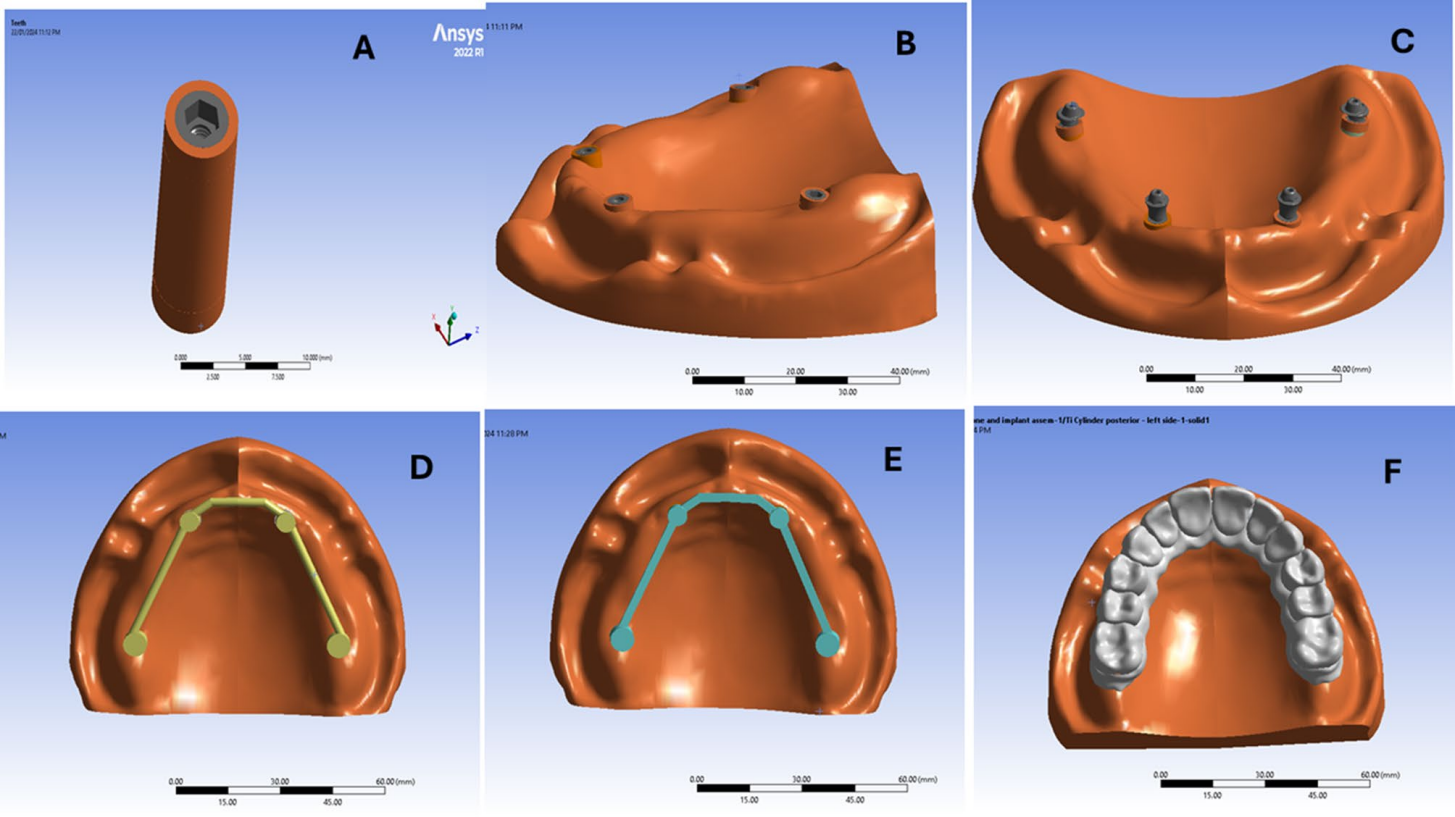
A photo showing the assembly of the implant into the bone cylinder (**A**), the assembly of the bone cylinder comprising the implant into the maxillary cast (**B**), the assembly of the straight multiunit abutment anteriorly and angled multiunit abutment posteriorly (**C**), the assembly of round cross section PEEK bar (**D**), the assembly of square cross section PEEK bar (**E**) and the assembly of PMMA superstructure (**F**) in the ANSYS software

The components were exported from the Solidworks software into the ANSYS16.2 software (Ansys, Inc, Pennsylvania, USA) program and presented as a function of area (Fig. 6). The ten node tetrahedral element was the element of choice used. Elastic modulus and Poisson’s Ratio were defined for each component. For nonlinear static analysis, a coefficient of friction (0.2) was given between the implants and the prosthetic superstructures [29, 31].The properties are listed in Table 1 [29, 31].

**Table 1:** Properties of each component in the model

Element	Poisson’s ratio	Modulus of elasticity (MPa)
Compact bone	0.3	15,000
Cancellous bone	0.3	1,500
PMMA	0.35	2,770
Titanium Alloy (Ti-Al6-V4)	0.3	110,000
PEEK	0.38	4,200

MPa: Mega Pascal unit

**Fig. 7:**
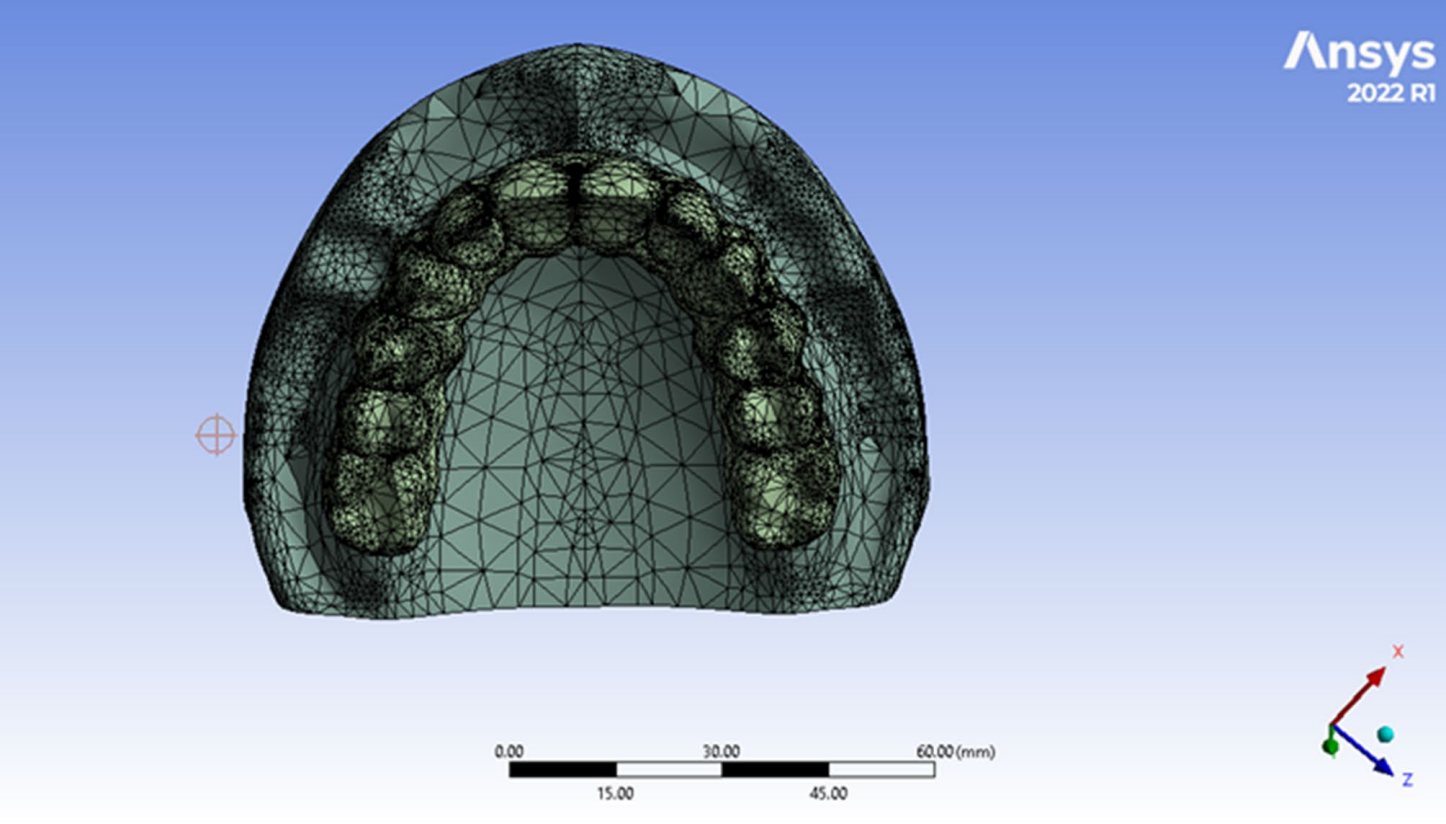
The process of meshing of the 3D model

The implant, multi-unit abutment and titanium cylinders were assumed to be made from titanium alloy. Both Rounded and Square bars were assigned to be made from PEEK. While the prosthetic superstructure was assumed to be made from Polymethylmethacrylate. All model materials were isotropic and homogenous (Fig. 7). The ten node tetrahedral element, element type solid 187 in ANSYS with element size 0.2 mm in the periimplant region was used. However, “adaptive mesh sizing” was adopted to ensure that the mesh is refined in regions of high stress or geometric complexity. Simulations with progressive finer mesh were made until the results got stabilized and further mesh refinement did not change the results significantly. The total number of elements in the model RB was 401,170, and in the model SB was 394,615. However, the number of nodes in the model RB was 686,785, and in the model SB was 676,117. Fixed restraints were applied to the inferior aspect and to all the laterals aspects of the maxillary virtual cast to avoid any bodily displacement during loading.

**Fig. 8:**
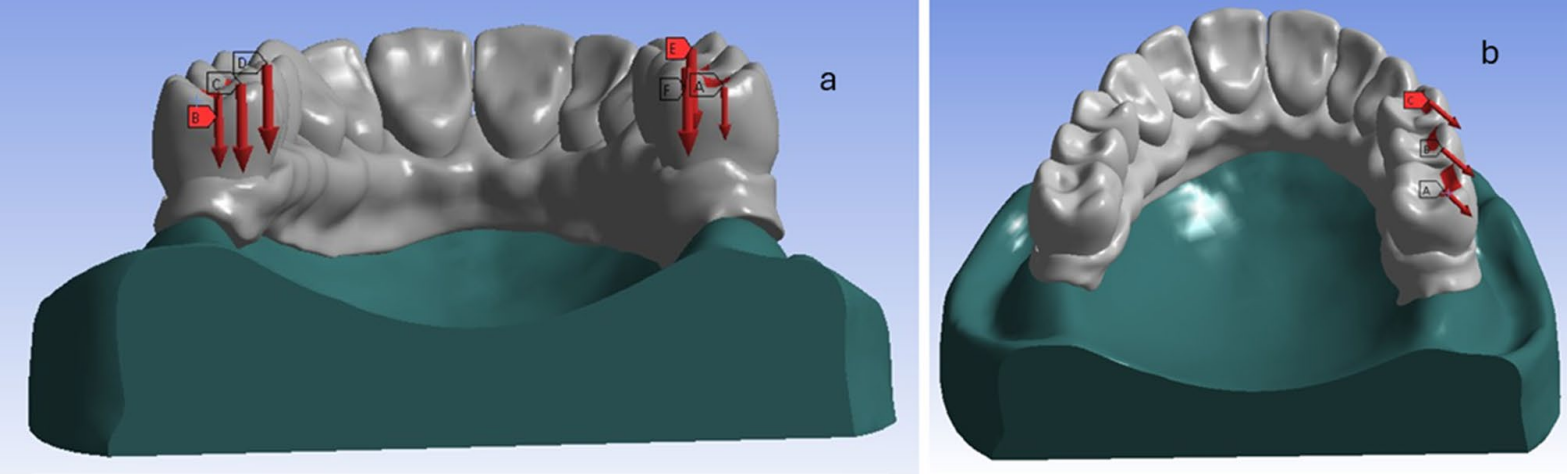
A photo showing vertical (**a**) and oblique (**b**) load simulation

Each model was subjected to two types of loads: vertical load and oblique load. For vertical load simulation, a total load of 200 N was applied bilaterally in the posterior region: 100 N load on the mesial marginal ridge and the central fossa of the first molar tooth and 50 N load on the mesial marginal ridge of each premolar (Fig. 8a). While for the oblique load, (Fig. 8b). a total load of 200 N was applied unilaterally in the posterior region to the lingual inclines of the buccal cusps of the premolar and first molar with an angle of about 45 degrees to the vertical axis of the tooth: 100 N load on the first molar tooth and 50 N load on each premolar [23, 25].

Each model was analyzed with the same exact boundary conditions and load application. The stresses displayed in this study are the von-Mises stress (SEqv) for the implants, marginal part of the bone cylinder, PEEK bar, prosthesis and the entire model. They were displayed as a graphical output in the form of a color-coded map and a numeric output that displayed the amount of maximum stresses in Megapascal (Mpa). The average stress value for each component was calculated. Calculation was made by summation of the stress values recorded in each cell the mesh constituting each component followed by division upon the number of cells [23, 25].

## Results

**Fig. 9:**
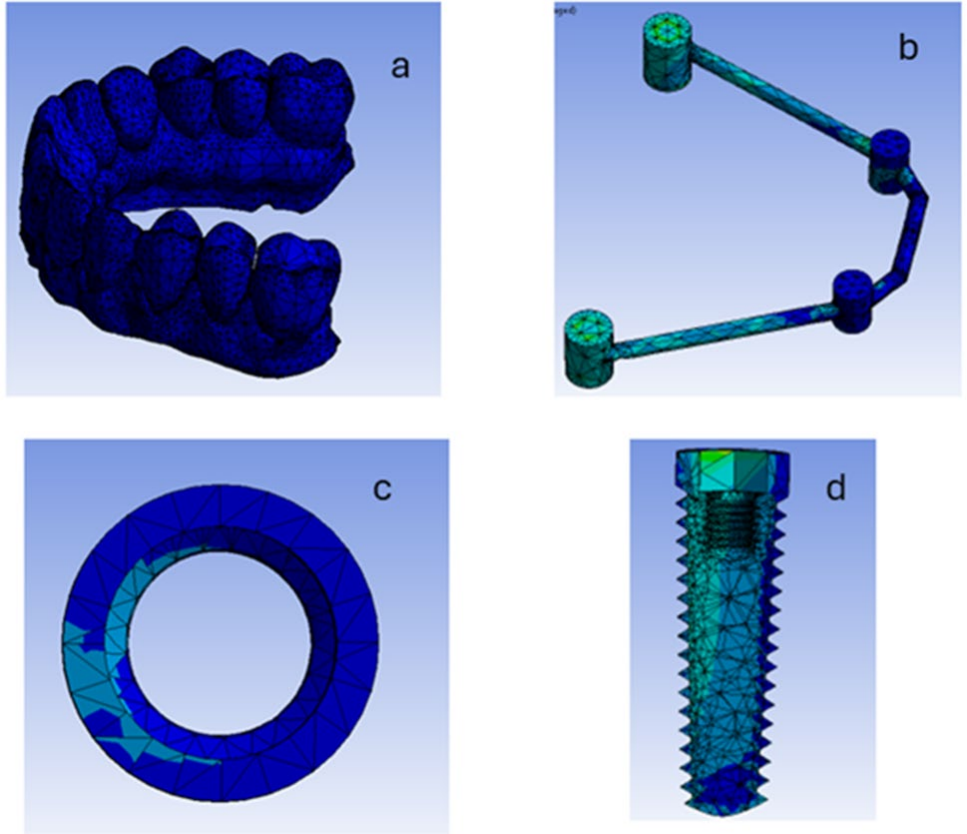
The stress distribution in the overlying prosthesis (**a**). the PEEK bar (**b**), the Peri-implant bone (**c**) and in the dental implant (**d**) during bilateral vertical loading scenario in the model RB

**Fig. 10:**
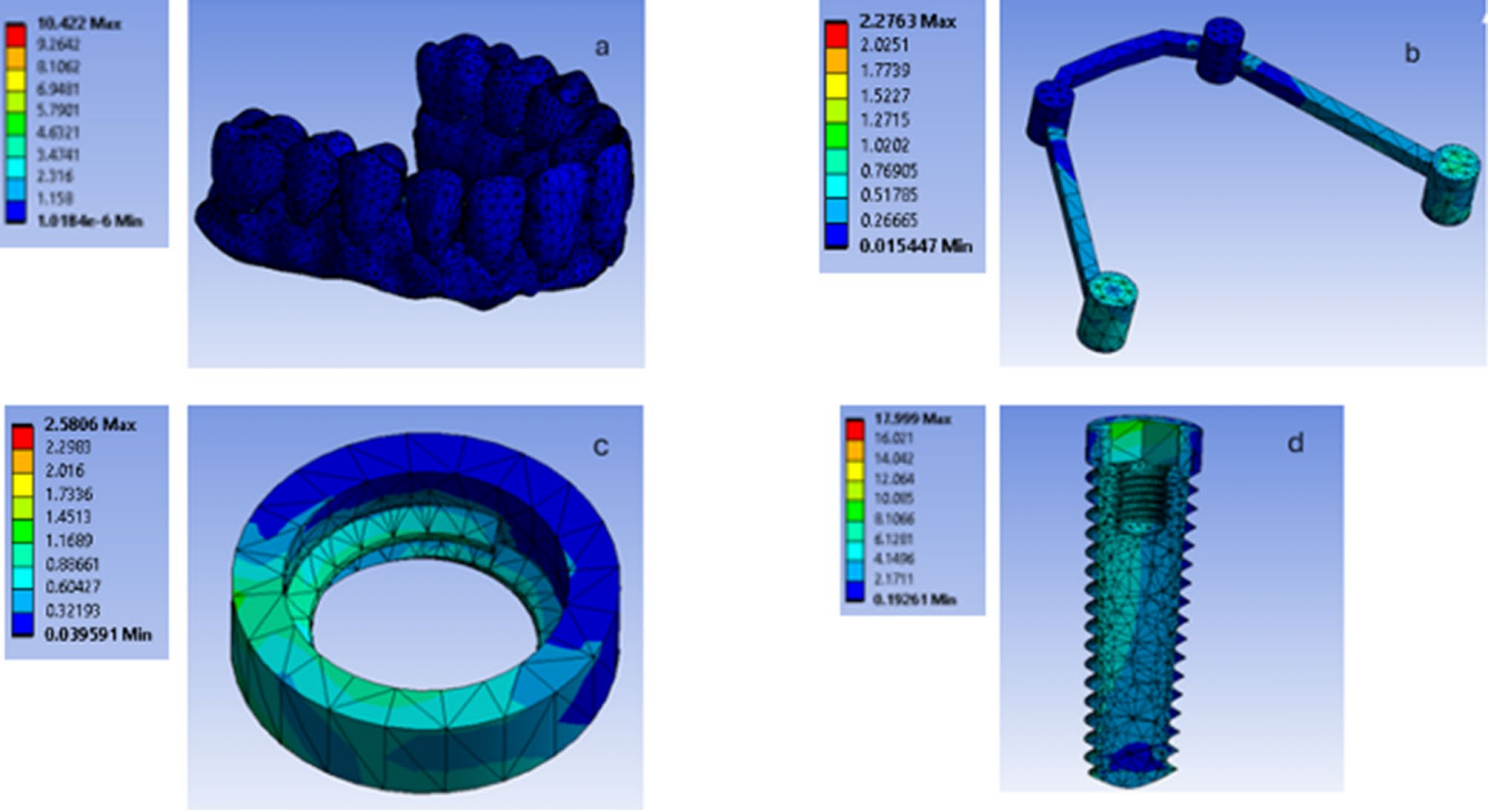
The stress distribution in the overlying prosthesis (**a**). the PEEK bar (**b**), the Peri-implant bone (**c**) and in the dental implant (**d**) during bilateral vertical loading scenario in the model SB

**Fig. 11:**
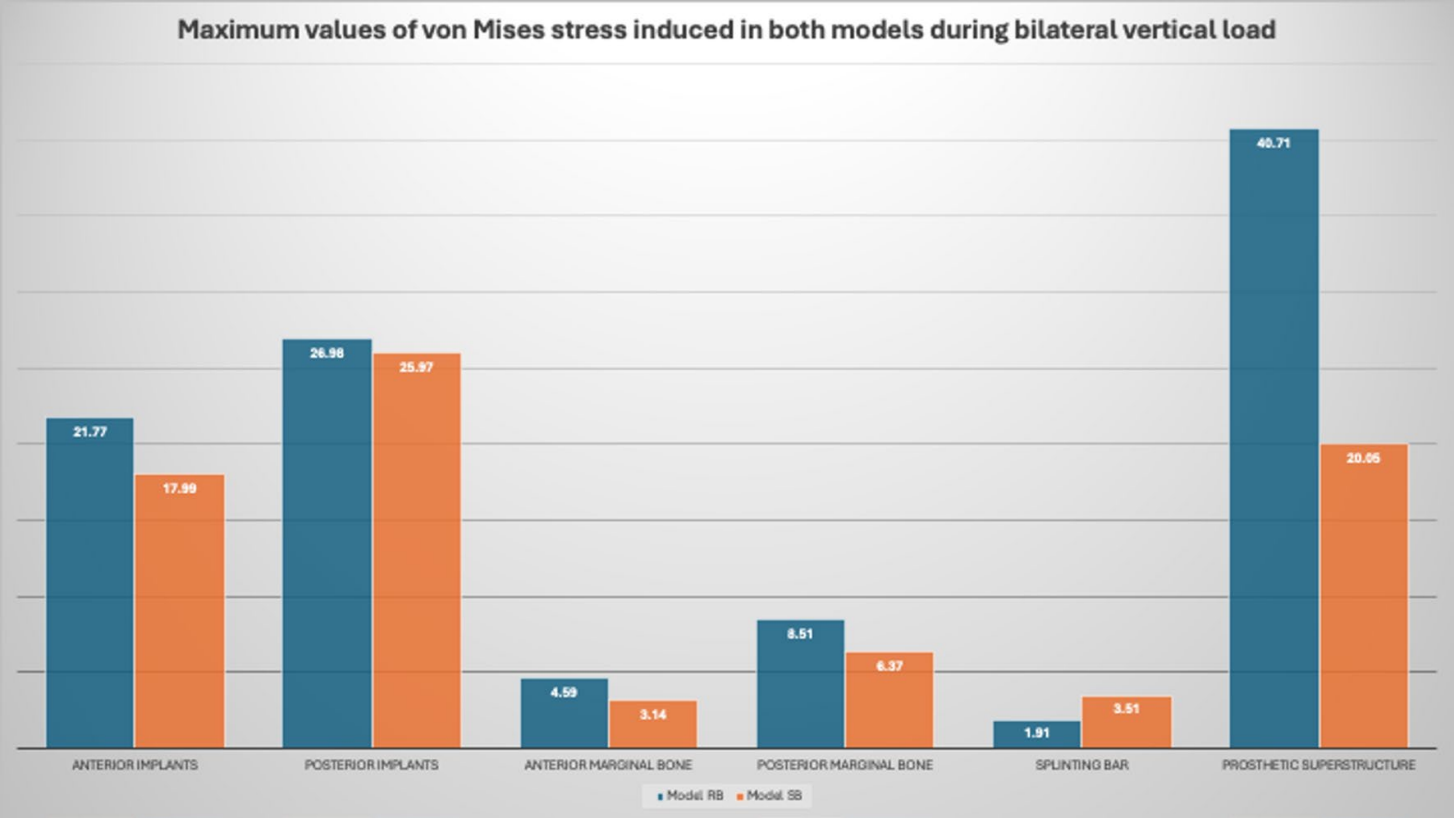
The maximum values of the von Mises stress induced in the implants, bone, bar and overlying prosthetic superstructure in both models during bilateral vertical loading

During bilateral vertical loading scenario in both models (the model RB and the model SB) the stress distribution in in the PEEK recorded the highest values posteriorly while in the Peri-implant bone the highest values recorded posteriorly in the coronal part and in the dental implant the highest values recorded posteriorly in the coronal part of the implant. (Figures 9, 10 and 11). 

For the stresses induced during vertical loading, stress concentration was observed in the posterior implants rather than the anterior ones in both virtual models. The posterior part of the PEEK bar as well received higher stresses than the anterior one. Moreover, it was more concentrated in the marginal area of the peri-implant bone rather than other areas of the supporting bone. Higher stress values were observed in the model RB compared to the model SB in the implants, peri-implant bone and overlying prosthetic superstructure. However, higher stress values were recorded for the splinting bar in the model SB compared to the RB one. The maximum values of the von Mises stress induced in the implants, bone, bar and overlying prosthetic superstructure in both models during loading are listed in Table 2 and the average values are listed in Table 3.

**Table 2:** The maximum values of the von Mises stress induced in the implants bone bar and overlying prosthetic superstructure in both models during loading

	Loading scenario	Model RB	Model SB
Anterior implants	Bilateral vertical load	21.77 MPa^*^	17.99 MPa^*^
	Unilateral oblique load	38.01 MPa^*^	33.4 MPa^*^
Posterior implants	Bilateral vertical load	26.98 MPa^*^	25.97 MPa^*^
	Unilateral oblique load	118.9 MPa^*^	110.51 MPa^*^
Anterior marginal bone	Bilateral vertical load	4.59 MPa^*^	3.14 MPa^*^
	Unilateral oblique load	20.69 MPa^*^	17.81 MPa^*^
Posterior marginal bone	Bilateral vertical load	8.51 MPa^*^	6.37 MPa^*^
	Unilateral oblique load	103.6 MPa^*^	100.29 MPa^*^
Splinting bar	Bilateral vertical load	1.91 MPa^*^	3.51 MPa^*^
	Unilateral oblique load	12.12 MPa^*^	20.94 MPa^*^
Prosthetic superstructure	Bilateral vertical load	40.71 MPa^*^	20.05 MPa^*^
	Unilateral oblique load	120.26 MPa^*^	97.68 MPa^*^

MPa: Mega Pascal unit, * symbol indicates von Mises stress

**Table 3:** The average values of the von Mises stress induced in the implants, bone, bar and overlying prosthetic superstructure in both models during loading

	Loading scenario	Model RB	Model SB
Anterior implants	Bilateral vertical load	1.13 MPa^*^	1.02 MPa^*^
	Unilateral oblique load	6.39 MPa^*^	6.21 MPa^*^
Posterior implants	Bilateral vertical load	4.21 MPa^*^	4.04 MPa^*^
	Unilateral oblique load	20.16 MPa^*^	20.04 MPa^*^
Anterior marginal bone	Bilateral vertical load	0.39 MPa^*^	0.37 MPa^*^
	Unilateral oblique load	2.06 MPa^*^	2.045 MPa^*^
Posterior marginal bone	Bilateral vertical load	0.609 MPa^*^	0.541 MPa^*^
	Unilateral oblique load	3.76 MPa^*^	3.64 MPa^*^
Splinting bar	Bilateral vertical load	0.285 MPa^*^	0.32 MPa^*^
	Unilateral oblique load	0.74 MPa^*^	0.83 MPa^*^
Prosthetic superstructure	Bilateral vertical load	0.32 MPa^*^	0.31 MPa^*^
	Unilateral oblique load	0.89 MPa^*^	0.882 MPa^*^

MPa: Mega Pascal unit, * symbol indicates von Mises stress

**Fig. 12:**
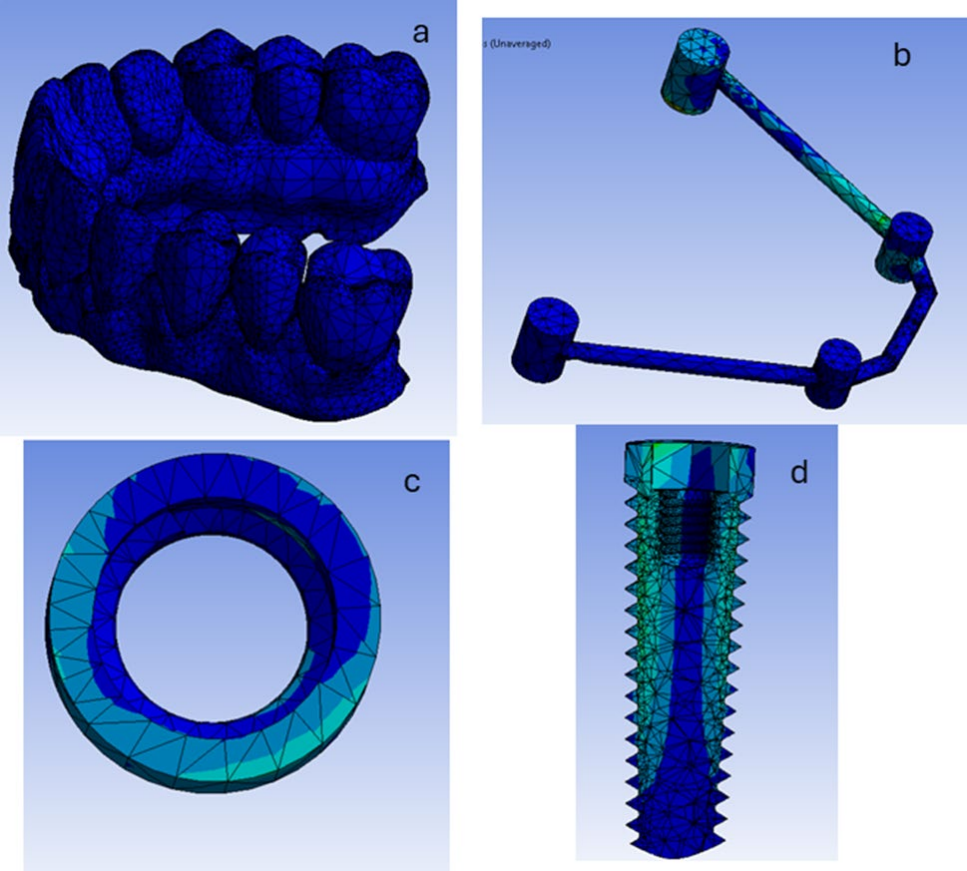
A photo showing the stress distribution in the overlying prosthesis (**a**), THE PEEK bar (**b**), the peri implant bone (**c**) and the dental implant (**d**) during the unilateral oblique loading scenario in the model RB

**Fig. 13:**
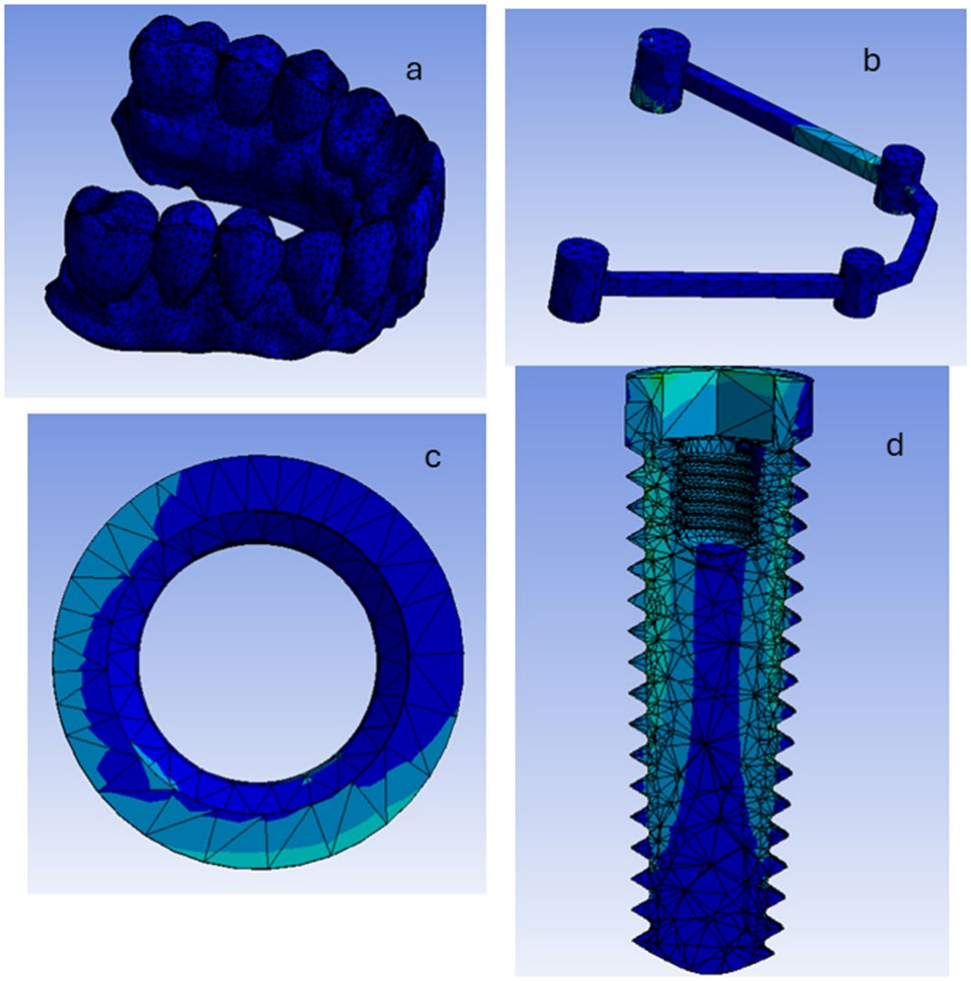
A photo showing the stress distribution in the overlying prosthesis (**a**), THE PEEK bar (**b**), the peri implant bone (**c**) and the dental implant (**d**) during the unilateral oblique loading scenario in the model SB

**Fig. 14:**
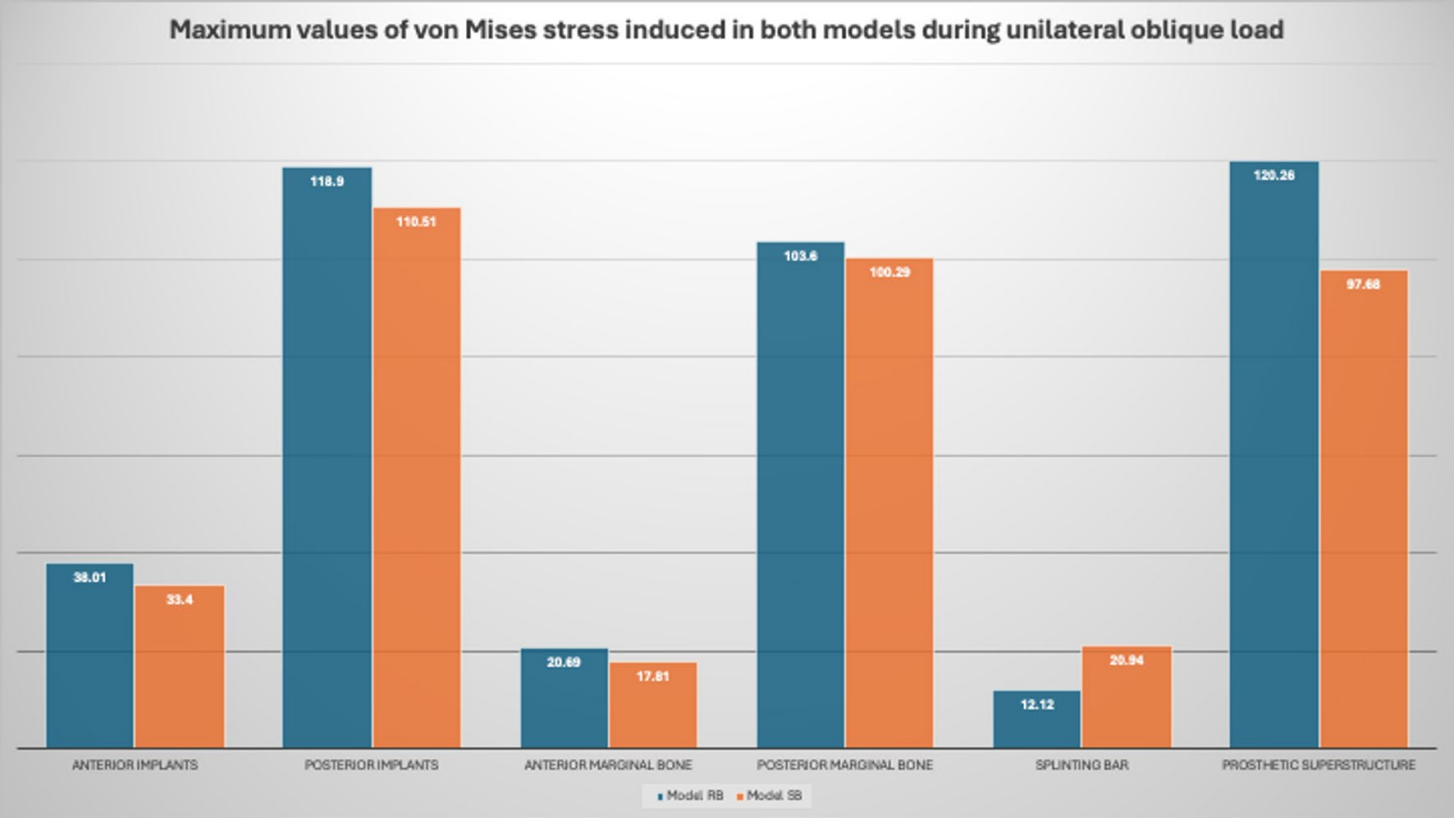
The maximum values of the von Mises stress induced in the implants, bone, bar and overlying prosthetic superstructure in both models during unilateral oblique loading

During the unilateral oblique loading scenario in both models (the model RB and the model SB**)** The stress distribution in the PEEK bar recorded the highest values in the left side. In the peri-implant bone the highest values recorded in the crestal region and in the dental implant the highest values recorded posteriorly in the coronal part of the implant. (Figs. 12, 13 and 14).

Regarding the stresses induced during oblique loading in the supporting bone, the highest value of the von Mises stress was recorded in the left side of both virtual models. Stress concentrations were observed in the posterior region of the models for the implants, splinting bar and peri-implant bone rather than the anterior one. Moreover, it was more concentrated in the marginal area of the peri-implant bone rather than other areas of the supporting bone as well as the coronal part of the dental implant. The implants, peri-implant bone and overlying prosthetic superstructure showed higher stress values in the model RB compared to the model SB. However, higher stress values were recorded for the splinting bar in the model SB compared to the RB one. The maximum values of the von Mises stress induced in the implants, bone, bar and overlying prosthetic superstructure in both models during loading are listed in Table 2 and the average values are listed in Table 3 (Figs. 13 and 14). Regarding the loading scenario, oblique loading also induced higher stresses compared to the vertical one in both models.

## Discussion

The purpose of the current study was to find out the difference regarding the stresses induced by the square and round cross sections of the PEEK splint bar supporting the provisional PMMA restoration in the All-on-4^®^ implant concept using Finite Element Analysis. The null hypothesis was rejected as higher stress values were observed in the model RB compared to the model SB in the implants, peri-implant bone and overlying prosthetic superstructure.

Finite element analysis (FEA) is a reliable method used to evaluate stress around the bone and the implants. This analysis generates computational data that reveal the behavior of new materials under simulated clinical conditions. Finite Element Analysis was used by several studies comparing hybrid prosthesis types, All-on-4^®^ concept and the superstructure materials [32–34].

In the current study, the All-on-4^®^concept was chosen as it is a reliable treatment option that was initially presented by Dr. Malo in 2003, that eliminates the necessity for bone augmentation. In addition to improving the load distribution, the angulation of distal implants creates an opportunity for the insertion of longer implants [3, 35] The distal implant was angulated at a 45-degree angle to be consistent with an earlier Finite Element Analysis study [36]. It is believed that the distal implant angulation has a major impact on lowering the strains at the cancellous and cortical bones. A study that examined the impact of various implant inclinations in the maxillary arch found that as distal implant angulation increased, the crestal bone stresses decreased. However, these results were not in agreement with the study conducted by Malhotra et al. who compared the stresses developed at 30 degree and 40 degree and found no statistically significant difference [37, 38].

PEEK was introduced as a splinting material. Recent suggestions to incorporate polymers into All-on-4^®^ concept aim to enhance patient satisfaction and confidence, addressing challenges associated with prosthesis fractures and failures [39].

According to previous study the occlusal masticatory forces in the tooth’s posterior region are approximately 220 N. Each model was subjected to two types of loads: vertical load and oblique load. For vertical load simulation, a total load of 200 N was applied bilaterally in the posterior region, while for the oblique load, a total load of 200 N was applied unilaterally in the posterior region. This was in accordance with previous studies [32, 40–42].

The loads applied in the current study were based on previous studies. Nonlinear stress analysis was followed as well, and the coefficient of friction was set to 0.2 to simulate a real condition. Furthermore, the von Mises stress was used to display the results as it is the most used value to evaluate the yielding behavior of the materials [29, 31].

Higher stress values were recorded posteriorly rather than anteriorly in both models. The posterior part of the PEEK bar as well as the implants received higher stresses than the anterior one. Such a result can be explained in the light of the fact that the posterior implants lie in the center of the masticatory region where most of the load is applied. Distal inclination of the posterior implants could be a further reason. The maximum value of the von Mises stress was recorded in the crestal bone and coronal region of the dental implants on the loaded side in both models during both loading scenarios. The results of the current study matched the results of several studies performed on the All-on-4^®^ implant supported prosthesis as that published by Homossany et al., Mohamed et al. and Güzelce et al. [29, 31, 43].

In current study the null hypothesis was rejected as in the model RB, higher stress values were recorded in the dental implants, peri-implant bone as well as the overlying PMMA provisional restoration compared to the model SB. Meanwhile, the PEEK splint bar received higher stress values in the model SB compared to the RB one. Such a finding can be explained in the light of the study that was conducted by Wang B et al., in which the higher rigidity of the square cross sectioned bar was found compared to the round cross sectioned. In that study, the mechanical behavior of both round and square cross sections were examined, and it was found that square cross section had higher efficiency (higher ability per unit area) in resisting bending and minimizing flexural deformation than the circular one. Additionally, circular cross section exhibited load–deflection responses [44]. Similarly, De Giorgis et al. stated that the more rigid the implant supported framework, the less stress delivered to the underlying implant. He reached a finding that a higher stiffness of the splinting bar material led to a smaller deformation of the prosthesis, thereby resulting in a better distribution of occlusal forces among the supporting implants. And this might also reduce the risk of fatigue and possible failures due to overloading of the implant prosthodontic components [45].

However, Nogueira et al. [46] and Dos Santos et al. [47] reported less stress values with rounded cross section bars compared to the oval and Hader ones. This could be attributed to the nature of the overlying prosthetic superstructure used in their study; an overdenture that undergoes more rotational movement with rounded bar compared to other cross sections leading to less stress delivered to the underlying implants. On the other hand, in the current study a fixed provisional prosthetic superstructure was used with no rotational movements taking place. Thus, the stress will be borne by the bar and the implants. As the bar is less rigid in the model RB compared to the model SB, more stresses were delivered to the underlying implants in the model RB.

De la rosa et al. evaluated the effect of different bars configuration on the implant supporting structures and stated that the bar cross section shape seems to affect stresses around implants. The stress transferred in the bone for a rectangular profile is higher than the round, L-shape and square ones. Except for the rectangular shape that had less material and cross-sectional area, modifying the shape of the bar had little influence on the levels of stress and strain transferred to the bone [48].

De carvalho also compared the mechanical behavior different designs of PEEK bars and found that the rectangular solid bars showed better compression strength than T-type and inverted T‐type bars, which are higher in resiliency and in a clinical scenario, it would result in a more effective dissipation of mastication loads. They attributed this result to the smaller contact area of the inverted T‐type creating a point where the stress is concentrated. They stated that PEEK should be considered for solid designs as it combines a high compression strength and degree of load absorption that make it suitable for a good biomechanical behavior of the prosthetic structure [49].

The stress values recorded during oblique loading were greater than the ones recorded during the vertical loading. Similar conclusions were reported by Holmgren et al., Jaros et al. and Bataineh et al. [30, 50, 51]. Such a finding may be contributed to the fact that the bone tissue was proven to be more resistant to the compressive loads, and less resistant to the tensile and shear loads [52].

In the clinical practice, the use of a square cross sectioned bar may reduce the stress delivered to the supporting implants helping to reduce the risk of biological and mechanical complications that may affect the process of implant osseointegration during the healing period. The same cross section may also help to reduce the stress induced in the overlying PMMA provisional restoration, thus reducing the risk of its fracture.

Despite the standardization and variables control in Finite Element Analysis studies, study limitations do exist. The stresses experienced by the implants and prosthesis in real conditions may have been underestimated in the current study as static loading was applied. However, dynamic loading is evident during chewing. Moreover, consistent living tissue simulation was not available as the material properties were assumed to be linearly elastic and isotropic. In addition, the geometry of the model components may have been simplified during modelling to overcome technical limitations related to the computer processors. Moreover, further clinical trials comparing the clinical outcomes as components fracture, screw loosening and loss of osseointegration between both bar designs should be performed.

## Conclusion

Within the current study limitations, Square cross sectioned Poly Ether Ether ketone bar induce less stress in All-On-4^®^ Implant Supported Interim Prosthesis compared to the round one.

## Data Availability

The data of the maximum stress values are included in the published article. The dataset used for calculation of the average stress value are available from the corresponding author upon request.

## Abbreviations

STL: Standard tessellation language
SEqv: Equivalent stresses; Von-Mises stress
N: Newton
MPa: Mega Pascal
